# Multi-Parametric Portfolio to Assess the Fitness and Gonadal Maturation in Four Key Reproductive Phases of Brown Trout

**DOI:** 10.3390/ani11051290

**Published:** 2021-04-30

**Authors:** Diana Santos, Eduardo Rocha, Fernanda Malhão, Célia Lopes, José F. Gonçalves, Tânia V. Madureira

**Affiliations:** 1 Interdisciplinary Centre of Marine and Environmental Research (CIIMAR/CIMAR), University of Porto (U.Porto), Terminal de Cruzeiros do Porto de Leixões, Av. General Norton de Matos s/n, 4450-208 Matosinhos, Portugal; dianapss@live.com.pt (D.S.); erocha@icbas.up.pt (E.R.); fcmalhao@icbas.up.pt (F.M.); celiacristinalopes@gmail.com (C.L.); jfmg@icbas.up.pt (J.F.G.); 2 Laboratory of Histology and Embryology, Department of Microscopy, Institute of Biomedical Sciences Abel Salazar (ICBAS), University of Porto (U.Porto), Rua Jorge Viterbo Ferreira 228, 4050-313 Porto, Portugal; 3 Department of Aquatic Production, Institute of Biomedical Sciences Abel Salazar (ICBAS), University of Porto (U.Porto), Rua Jorge Viterbo Ferreira 228, 4050-313 Porto, Portugal

**Keywords:** blood lipids, hormones, immunohistochemistry, testis, ovary, reproductive cycle, *Salmo trutta*

## Abstract

**Simple Summary:**

Brown trout is a freshwater fish with economic importance and with a great potential to be used as an environmental biosensor species. Despite being selected as a model species in distinct scientific contexts, in cultured specimens, there is a surprising lack of works investigating the morpho-physiological changes associated with the reproductive cycle; particularly concerning the gonads. In this study, a multi-parameter portfolio of biometric, biochemical, hormonal, and morphological analysis was established, which allowed a seasonal and sex characterization of the gonad status of adult brown trout males and females. Sampling included four reproductive phases: spawning capable (December), regressing (March), regenerating (July), and developing (November). Sex- and season-specific changes were described. The discriminative parameters characterized here stand now as normal baseline values against which abnormal patterns can be compared with. These parameters have the potential to be used as tools for the environmental monitoring of the reproductive status of wild populations and for the control of breeding stocks in aquaculture.

**Abstract:**

Brown trout is an environmental freshwater sentinel species and is economically important for recreational fishing and aquaculture. Despite that, there is limited knowledge regarding morpho-physiological variations in adults throughout the reproductive cycle. Thus, this study aimed to analyze the fitness and gonadal maturation of cultured adult brown trout in four reproductive phases (spawning capable—December, regressing—March, regenerating—July, and developing—November). The systematic evaluation of males and females was based on biometric, biochemical, and hormonal parameters, along with a histomorphological grading of gonads and the immunophenotype location of key steroidogenic enzymes. The total weight and lengths reached the lowest levels in December. Gonad weights were higher in December and November, while the opposite pattern was found for liver weights. The lowest levels of cholesterol and total protein were also noted during those stages. The 11-ketotestosterone (11-KT) and testosterone (T) for males, and estradiol (E2) and T for females, mostly explained the hormonal variations. The immunohistochemistry of cytochrome P450c17 (CYP17-I), aromatase (CYP19), and 17β-hydroxysteroid dehydrogenase (17β-HSD) showed sex and site-specific patterns in the distinct reproductive phases. The sex- and season-specific changes generated discriminative multi-parameter profiles, serving as a tool for environmental and aquaculture surveys.

## 1. Introduction

In general, fish have high adaptability to cope with seasonal cycle changes, specifically those related to gonad development and maturation, which are highly correlated to prevailing climatic conditions, including temperature and photoperiod [1]. Regarding salmonids, seasonal monitoring studies have shown that blood levels of a few hormones, such as the sex-steroids testosterone (T), estradiol (E2), and 11-ketotestosterone (11-KT), are high before spermiation and ovulation, and so may indicate that the maturation stage is reached [2,3,4]. T is a male-specific androgen that also functions as a precursor for E2 synthesis [5], which subsequently induces the synthesis of hepatic vitellogenin (Vtg) during the process of vitellogenesis that occurs physiologically in females [6]. As for 11-KT, it is mainly associated with spermatogenesis, but there is also evidence of seasonal fluctuations in females [3,4]. Additionally, seasonal changes along the reproductive cycle of salmonids have been described for pituitary gonadotropins, which induce the production of steroid hormones in the gonads. In female rainbow trout (*Oncorhynchus mykiss* Walbaum, 1792), the follicle-stimulating hormone (FSH) increases progressively through oocyte growth and at ovulation [7], and the luteinizing hormone (LH) peaks specifically during maturation [8]. In female brown trout (*Salmo trutta* Linnaeus, 1758), 17-hydroxyprogesterone (17-OHP), a steroid involved in the production of T, showed higher levels at ovulation [9]. Several other markers, such as the biochemical ones, have also been shown to follow a seasonal pattern in salmonid reproductive cycles. Total plasmatic calcium, correlated with Vtg levels, peaks at vitellogenesis [10], while blood cholesterol was higher during the early stages right after ovulation [11].

Brown trout is a highly valued species for sport, fishery, and aquaculture [9,12,13]. Brown trout have a wide geographical distribution throughout Europe, North Africa, and western Asia, stretching from Norway in the northwest to the Aral Sea in the east. Since the mid-19th century, brown trout have been widely introduced into suitable environments worldwide, including North and South America, Australasia, Asia, and South and East Africa [13]. Brown trout are characterized by having an extremely plastic life history, occurring as either anadromous (sea trout) or non-anadromous forms, and maturing from any age between 1 and 10 years. Brown trout has a very high genetic variation and a wide range of morpho-phenotypes. Specific populations may differ from each other in molecular genetic markers, color, size, and seasonality [14,15]. For instance, specimens from an Iberian Peninsula wild population reached 39.5 cm in length and around 1 kg in weight [16], while sexual maturity occurred at any age between 1 and 10 years [13]. The brown trout is increasingly being recognized as a good bioindicator species, due to both its wide geographical distribution and its low tolerance to water quality degradation, in comparison, for instance, with rainbow trout [17].

Despite the high economic and ecotoxicological interest of brown trout, the seasonal characterization of its reproductive cycle is surprisingly limited [18,19,20]. In the eighties, Billard [21] performed a detailed quantitative description of the spermatogenesis and oogenesis in (14-month-old) brown trout farmed in the United Kingdom over a 20-month period. Likewise, cultured (3-year-old) brown trout females in Chile were characterized based on oocyte growth, gonad histology, and plasma levels of key sex-steroid hormones along a reproductive cycle [9]. Details of the reproductive cycle have also been reported in natural brown trout populations. For instance, animals from the Yadong River in Tibet, ranging from 1 to 4 years old, were monitored to evaluate several parameters, including the age at sexual maturity, fecundity, egg size, and spawning season, which occurred mainly in November and December [22]. Accurate investigation of the reproductive phases of brown trout and its relationship with the histomorphology of the gonads and with the biochemical and hormonal profiles could reveal itself as a useful tool to sustain either environmental or aquaculture-related applications. These kinds of studies are still lacking in brown trout from the Iberian Peninsula.

In this study, we aimed to analyze four phases of the reproductive cycle of a cultured population of adult (3-year-old) brown trout from both sexes. Further, the animal’s fitness condition was also assessed by checking key parameters at the distinct samplings. The four phases are representative periods of the year, in which brown trout would reach the main reproductive stages in this geographic area (North of Portugal) and were sampled according to one established nomenclature [23]: December, spawning capable; March, regressing; July, regenerating; and November, developing. Following this classification, in July the predominant germ cells in the gonads of both sexes are immature (spermatogonia and oogonia in males and females respectively), while by December gametogenesis is well advanced with the gonads containing mature germ cells. For each reproductive stage, a vast set of plasma, blood, or serum hematologic and biochemical parameters (hemoglobin, cholesterol, triglycerides, glucose, and potassium, among others) and six hormones (FSH, LH, 17-OHP, 11-KT, E2, and T) were analyzed. Additionally, to evaluate the gametogenic stages in each sampled phase, a qualitative and quantitative histomorphological evaluation of male and female gonads was made. The cytohistological location of cytochrome P450c17 (CYP17-I), aromatase (CYP19), and 17β-hydroxysteroid dehydrogenase (17β-HSD)—three of the main steroidogenic enzymes in brown trout gonads—was assessed by immunohistochemistry. In the end, we made a step forward by statistically defining from the tested parameters those that had the strongest patterns of variation in cultured brown trout during the four phases of the reproductive cycle. It is already known that differences do exist between cultured and wild specimens, but the proposal here is to define a selection of parameters that can be explored as an effective tool for both environment and aquaculture monitoring of trout gonadal status and as a starting point to implement in other fish species.

## 2. Methods

### 2.1. Study Species

Adult brown trout (*Salmo trutta*) were born and held in captivity within three spawning cycles (≈3-year-old) in the Torno Aquaculture Station (Amarante, Portugal). Trout stock is regularly renewed, with the introduction of wild animals, in order to promote crossbreeding and avoid inbreeding. Specimens were being maintained in pristine water supplied by the natural mountain course of Ribeiro do Ramalhoso, under a continuous through-flowing system. Fish were kept under natural photoperiod, in race-way tanks with approximately 12 × 7 × 2 m^3^ (length, width, and height), which hold an average of 400 to 500 adult trout (males and females). Six animals per sex were randomly sampled from rearing tanks in December 2017, March, July, and November 2018, thus including four phases of gonadal maturation along a natural reproductive cycle. Animals were fed daily with trout food pellets (ICNF7MM, AQUASOJA), except on the day before sampling. The handling of animals followed the Portuguese Decree-Law No. 113/2013, which translates the EU Directive No. 2010/63 on animal protection for scientific purposes. To characterize the water conditions in the aquaculture facilities at the time of sampling, the temperature (°C) (VWR DO 210, Shanghai, China), pH (WTW pH 530, Weilheim, Germany), dissolved oxygen—O_2_ (%) (VWR DO 210, China), hardness (dGH) (PRODACtest TGH, Italy), nitrites—NO_2_ (mg/L) (PRODACtest TNO2, Cittadella, Italy), nitrates—NO_3_ (mg/L) (Sera Nitrate-Test, Germany), and ammonia—NH_3_ (mg/L) (PRODACtest TNH3.2, Italy) were measured locally.

### 2.2. Sampling Procedures

To limit eventual diurnal variability, animals were sampled within the same timeframe (10 a.m. to 4 p.m.). Euthanasia was performed using an overdose of an aqueous solution of ethylene glycol monophenyl ether (2 to 3 mL/L) (Merck, Darmstadt, Germany). Fish were weighed (g) with a digital dynamometer (Kern CH15K20, Balingen, Germany) and measured, considering the total and standard lengths (cm), to determine the condition factor (K), following the calculation: K = 100 × fish wet weight (g) ÷ (total length)^3^ (cm) [24].

Blood was collected through the caudal vein using 5 mL syringes (B.Braun Injekt, Germany) and 20 G needles (B.Braun Sterican 20 G × 1”, Germany), and transferred to tubes coated with lithium heparin (Vacuette LH Lithium Heparin, Austria) or ethylenediaminetetraacetic acid-EDTA (Vacuette K3EDTA, Austria), and manually shaken. Plasma was obtained after blood centrifugation at 2000× *g* for 20 min (VWRMicro Star 12, Denmark).

The liver and gonads were collected and weighted to calculate the organ-somatic indexes, as follows: organ weight (g) ÷ fish weight (g) × 100. For females, two to eight fragments per gonad with 1 to 2 cm were sectioned and used to determine the total number of eggs per gonad. In addition, 100 eggs/female were photographed using a stereomicroscope (Leica EX4W, Wetzlar, Germany) at 0.8× (December, July, and November) or 1.6× magnification (March), for diameter assessment using Image J software (version 1.51j8). Only eggs with a diameter higher than 0.151 mm were analyzed. In March and July, the dissociation of eggs was performed by using an enzymatic solution of 0.3% trypsin in phosphate-buffered saline (PBS), pH 7.2, at 37 °C, as previously described for gonad cell isolation in fish [25]. A gentle mechanic dissociation was also implemented whenever it was needed to help in the egg dissociation process.

### 2.3. Blood, Plasma, and Serum Quantifications

#### 2.3.1. Biochemical Analyses

Cholesterol (mg/dL), triglycerides (mg/dL), glucose (mg/dL), hemoglobin (g/dL), high-density lipoprotein cholesterol (HDL) (mg/dL), and potassium (mmol/L) were quantified with a Reflotron Plus equipment (Roche Diagnostics, Switzerland), using specific Reflotron reactive strips, according to the manufacturer’s recommendations. Those analyses were performed on total heparinized blood, except for HDL and potassium, which were measured in EDTA and heparinized plasma samples, respectively. Each of the Reflotron reactive strips analyses required a total of 32 μL of the sample. Low-density lipoprotein (LDL) cholesterol (mg/dL) was calculated with the Friedewald formula: LDL = total cholesterol − HDL − triglycerides ÷ 5.0 (mg/dL) [26], while the very-low-density lipoprotein (VLDL) was calculated by dividing the triglyceride value per 5 [27]. To guarantee the accuracy of the optical system, control measurements were routinely made using Reflotron Check strips. Whenever the values of the analyzed parameters were lower than the limit of quantification, a value immediately below the minimum possible to measure was considered for data analyses.

Other biochemical parameters were also determined in a certified laboratory (INNO-Serviços Especializados em Veterinária, Braga-Portugal), using serum samples from the same fish, including calcium (mg/dL), ionized calcium (mg/dL), phosphorus (mg/dL), sodium (mEq/L), chlorine (mEq/L), total protein (g/dL), and magnesium (mg/dL).

#### 2.3.2. Hormone Plasma Levels

Different commercial Enzyme-Linked Immunosorbent Assay (ELISA) kits were used to quantify the plasma hormone levels of FSH (abx051242, Lot No. L201804J147, Abbexa, UK), LH (abx051240, Lot No. L201804H375, Abbexa, UK), 17-OHP (abx257402, Lot No. L201804W675, Abbexa, UK), E2 (ADI-900-008, Lot No. 07141718A, Enzo, USA), T (ADI-900-065, Lot No. 12211719B, Enzo, USA), and 11-KT (582751, Lot No. 0505639, Cayman Chemical, USA). The analytical procedures were followed as indicated by the manufacturer. A total of 12 plasma samples (6 from each sex) were analyzed per sampling period. Heparinized plasma was used to quantify E2, T, and 11-KT levels, while for the analysis of FSH, LH, and 17-OHP, plasma EDTA samples were required. Both types of samples were preserved by deep freezing in liquid nitrogen and then stored at −80 °C. Plasma samples were diluted as follows: FSH—1:5, LH—1:5, 17-OHP—1:10, E2—1:10 or 1:50, T—1:20 (except for the females of July which were not diluted), 11-KT—1:50 for males of March, 1:100 for males of July, 1:1000 for males of November and December, or no dilution for all females. The detection limits for each kit were as follows: FSH—0.6 ng/mL, LH—0.08 ng/mL, 17-OHP—0.313 ng/mL, E2—29.3 pg/mL, T—7.81 pg/mL, and 11-KT—0.78 pg/mL. FSH, LH, and 11-KT kits have cross-reactivity in fish, while the remaining kits have a species-independent reactivity. Intra- and inter-assay coefficients of variation were overall below 10% and 15%, respectively. The recommended absorbance for each kit was measured in a microplate reader (Thermo Scientific Multiskan GO, Finland), and data calculations were made according to each kit specification.

### 2.4. Histological Analyses of the Gonads

#### 2.4.1. Paraffin Procedures

Gonad fragments from all animals (6 males and 6 females per sampling stage) were fixed in 10% buffered formalin for 48 h, at room temperature, and then transferred to 70% ethanol. Two random fragments of each of the sampled gonads were submitted to a 12 h routine histological protocol in an automatic processor (Leica TP 1020, Wetzlar, Germany), which included: ethanol 70%, ethanol 90%, ethanol 96%, ethanol 99.9%, ethanol 99.9%/xylene, xylene, xylene/paraffin, and paraffin. A longer (manual) protocol (23 days) was required for mature ovaries from December and November. The processed fragments were embedded in paraffin (Thermo Scientific Histoplast, UK) in an embedding center (Leica EG 1140 C, Germany). Two slides with two sections each, 3 μm thick and distancing 15 μm from the other two slides, were obtained per block with an automatic rotary microtome (Leica RM2255, Germany). Two slides were used for immunohistochemistry, and the other two for hematoxylin and eosin (H&E) staining.

#### 2.4.2. Methacrylate Procedures

Following fixation in 10% buffered formalin, one random fragment of gonad from 2 animals was chosen to represent the individual maturation stages of each sex. Subsequently, the selected fragments were manually processed using the Technovit H7100 Embedding Kit (Kulzer 14653, Germany). Steps included: (a) progressive dehydration with four series of ethanol (70%, 90%, 96%, and 99.9%, 1 h each); (b) pre-infiltration in a 1:1 mix of infiltration solution and 99.9% ethanol (2–4 h); (c) overnight infiltration with infiltration solution; (d) polymerization in proper molds and embedding at room temperature for 1 h, followed by an overnight period at 37 °C. All fragments were processed in a short protocol (33 h), except for ovaries with mature eggs, from December and November, which were handled using a protocol with longer incubation periods (dehydration and pre-infiltration stages lasted for 2 to 3 days, and the infiltration stage increased to 7 days, totalizing 20 days).

Blocks were sectioned (5 μm) in a standard microtome (Leica RM2155, Germany) using a tungsten carbide knife (Leica, Germany). Sections were stained using an adapted H&E staining protocol to evaluate gonadal maturation, while Sirius red technique [28] and reticulin staining [29] were used to specifically reveal collagen and reticular fibers. All slides were coated with silane to improve tissue adhesion. The H&E staining protocol consisted of placing the slides in Mayer’s hematoxylin for 30 min (Merck, Germany), washing the slides in water before 10 min in eosin (Merck, Germany), washing the slides in water again, and finishing with dehydration, clearing, and mounting (VRW Coverquick 2000, Fontenay-sous-Bois, France). Sirius red staining [28] consisted of covering the slides with celestine blue for 15 min; washing in distilled water for 5 min; staining the nuclei with Mayer’s hematoxylin (Merck, Germany) for 20 min; washing in distilled water for 5 min; staining with picro-Sirius red overnight at 23 °C (room temperature); washing twice in 0.5% acetic acid; washing in distilled water; dehydrating three times in 99.9% ethanol, 1 min each; clearing twice in xylene for 2 min each and mounting the slides (VRW Coverquick 2000, France). As for reticulin staining [29], the protocol included several stages: covering the slides with 0.75% potassium permanganate for 2 min; washing in tap water; washing with 10% potassium metabisulfite until the brown color disappears; washing with tap water for 10 min; covering with 2% ferric alum for 5 min; washing with tap water for 5 min; washing twice in distilled water; impregnation with ammoniacal silver for 90 s; washing in distilled water 5 to 10 times; reducing with 10% unbuffered formalin for 3 min; wash in tap water for 15 min; covering with 0.2% gold chloride for 8 min; washing with distilled water; covering with 1% sodium metabisulfite—1 min; covering with 1% sodium hyposulfite for 1 min; washing in tap water; contrasting with nuclear red for 15 min; dehydrating three times in 99.9% ethanol for 1 min each; clearing twice in xylene for 2 min each, and mounting the slides (VRW Coverquick 2000, France).

### 2.5. Qualitative and Semi-Quantitative Grading of Gonadal Maturation Stages

Gonad sections (*n* = 4, per gonad) from all animals were stained with H&E and observed in a light microscope (Olympus BX50, Tokyo, Japan), in order to grade male and female developmental stages, according to the Organization for Economic Co-operation and Development (OECD) criteria [30], which were here adapted for the species, establishing here five stages for males and females.

### 2.6. Immunohistochemistry

Two random fragments of each gonad (from 6 females and 6 males of each reproductive phase) were used for immunohistochemistry. From each of those fragments, 4 sections were obtained. After deparaffinization, sections were hydrated in a decreasing series of ethanol until water. For the antigen retrieval, slides were transferred into a pressure cooker with pre-warmed citrate buffer (0.01 M, pH 6.0), and left to boil for 2 min at maximum pressure. After cooling, the endogenous peroxidase was neutralized using a 3% hydrogen peroxide solution (Merck, Germany) in methanol (Sigma-Aldrich, France) for 5 min. Slides were then covered with the protein blocking solution of the Novolink Polymer Detection Systems (Leica Biosystems, UK) for 5 min. The incubation with the primary antibodies was performed overnight at 4 °C, in a humidified chamber, and the antibodies were diluted in PBS with 5% bovine serum albumin (BSA), as follows: CYP17a1 (CYP17-I) (donated by Hinfray, et al. [31]), diluted at 1:300; CYP19 (Santa Cruz Biotechnology, sc-374176), diluted at 1:20; and 17β-HSD (Santa Cruz Biotechnology, sc-373902), diluted at 1:50. The CYP17-I antibody was raised against two peptides of the zebrafish Cyp17a1 sequence, and at least one peptide showed high similarity with the *Salmo trutta* sequence. The CYP19 and 17β-HSD antibodies used were human-specific, because there are no available commercial antibodies for the corresponding fish proteins. Despite this, the epitopes are fairly conserved in the *Salmo trutta* sequence. The post-primary incubation (30 min) preceded the incubation with the Novolink polymer for 30 min. The 3,3′-diaminobenzidine (DAB) working solution was used as a chromogen, followed by the counterstaining with hematoxylin (Merck, Germany). Negative controls were performed in every slide, by incubating PBS with 5% BSA rather than the primary antibody. Slides were qualitatively analyzed under a light microscope (Olympus BX50, Japan) and photographs were obtained with a digital camera (Olympus DP21, Japan).

### 2.7. Statistical Analysis

The analysis was performed with STATISTICA software, version 13.4.0.14 for Windows, TIBCO Software Inc, and with Past software, version 3.26 for Windows [32]. Normality and homogeneity of variances were tested using Shapiro-Wilk’s normality test and Levene’s test, respectively. When required, data were transformed in logarithm, inverse, square root, square, or cube. If assumptions were still not achieved, data were transformed in ranks. As some data sets did not pass the normality test, we opted to use the median, maximum, and minimum in the descriptive statistics. Two-way ANOVA was made for all parameters, except for egg diameter, total egg number, GSI (gonado-somatic index), and gonad weight, where one-way ANOVA was applied (i.e., data were separated by sexes). Whenever significant, the analysis of variance was followed by the post-hoc Tukey test to determine differences between pairs. For the grading classification of female and male gonads, the parametric assumptions were not met, therefore, the significant differences were confirmed by using the non-parametric Kruskal Wallis ANOVA by ranks, followed by the Mann–Whitney pairwise test (with sequential Bonferroni correction). Significance was considered when *p* < 0.05. Principal component analysis (PCA) was implemented with PAST by using the correlation matrix for biometric, biochemical, and hormone parameters for each sex, explaining the maximum amount of variance with the least number of components. PCA of both biometric and biochemical parameters was performed excluding the redundant variables, and those that were calculated from other parameters, to reduce the number of variables. For the biometric parameters, we included the total weight, total length, liver weight, gonad weight, and condition factor. From the biochemical parameters, cholesterol, triglycerides, glucose, hemoglobin, hematocrit, total protein, phosphorus, sodium, chlorine, magnesium, potassium, and total calcium were included. To determine the total number of principal components, eigenvalues greater than 1 were selected.

## 3. Results

### 3.1. Physico-Chemical Parameters of Water

The mean values of the distinct water quality parameters are described in Table 1.

### 3.2. Biometric Measurements

Biometric data are detailed in Table 2. Total weight and lengths seemed lower in December and November, for both females and males, when compared to those obtained in March and July, although it was not possible to prove significant differences in all cases. The minimum value of total weight for females was 440 g in December, and the maximum was 2180 g in July. For males, the lightest specimen was sampled in November (with 580 g), and the heaviest (with 2040 g) in July. As for the total and standard lengths, the minimum values for females were observed in December (34.0 and 31.5 cm) and the maximum in July (54.5 and 50.0 cm). Males from December and November had significantly lower median lengths than those from July. The K values did not change along the seasons for the females, but males from December had significantly lower median K than fish from March. The lowest median GSI was noted in March for females (0.42%) and in July for males (0.29%). Contrarily, the highest medium GSI values were registered in November for both females (20.54%) and males (4.26%). Irrespective of the gender, brown trout from December and November had significantly higher gonad weight, compared to fish from March and July. For both sexes, liver weight was significantly lower in December than in March and July. However, no significant differences were noticed in the HSI values. HSI ranged from 0.68% to 2.07% (both in December) for females, and from 0.77% to 1.60% (both in July) for males.

The PCA plot of biometric parameters for females and males allowed for the discrimination of the four reproductive phases, with December and November, and March and July presenting closer and, therefore, overlapping patterns (Supplementary Figure S1). For males (Supplementary Figure S1A), the first factor account for a total data variation of 69.55%, and PC1 had higher positive associations with liver weight, total weight, and total length. For females (Supplementary Figure S1B), PCA extracted one factor, with an eigenvalue greater than 1, which explained 68.75% of the variation in the data. Total weight, total length, and liver weight had the higher positive loadings on PC1.

**Figure 1:**
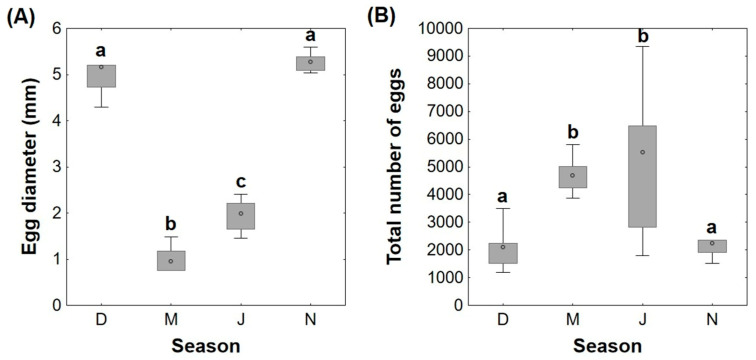
Egg diameter (mm) (**A**) and total number of eggs (**B**) in female adult brown trout gonads in distinct reproductive phases (December—D, March—M, July—J, and November—N). Data are given as median (circle in the box), 25% and 75% percentile (upper and lower limits of box), and maximum and minimum values (in the whiskers); *n* = 6 animals/season. Different lower-case letters represent significant differences (*p* < 0.05) between seasons.

The egg diameter was significantly higher in December (median 5.16 mm) and November (median 5.27 mm), compared to the other seasons (medians March—0.96 mm and July—1.99 mm) (Figure 1A). Also, eggs from March had significantly lower diameters than in July. In terms of egg quantity, there was a significantly lower number of eggs in December (median 2098) and November (median 2245) in comparison to March (median 4679) and July (median 5529) (Figure 1B).

### 3.3. Biochemical Analyses

Biochemical data are summarized in Table 3. For each sex, differences among seasons were again mostly noted between December/November and March/July. In terms of lipid parameters, for both sexes, the lowest medians of cholesterol, LDL, and cholesterol/HDL ratio were measured in December and November. For those parameters, the highest medians were obtained in March, differing significantly from December and November. HDL was always higher than 100 mg/dL. Triglycerides (minimum and maximum values for females—85.2 to 168.0 mg/dL and males—79.5 to 125.0 mg/dL) and VLDL levels (minimum and maximum values for females—17.0 to 33.6 mg/dL and males—15.9 to 25.0 mg/dL) did not show significant differences among the different seasons.

As for non-lipidic parameters in both sexes, glucose concentrations were the highest in March and the lowest in November, for females, and in December, for males. Overall, hemoglobin levels were almost always below the limit of quantification, and hematocrit was a quite stable parameter. The total protein levels in females from December (median 3.7 g/dL) and November (median 3.4 g/dL) were significantly lower than in March (median 5.2 g/dL) and July (median 5.8 g/dL). Regarding the males, December (median 3.7 g/dL) also had significantly lower values when compared to March (median 5.3 g/dL) and July (median 4.6 g/dL). No significant variations occurred for total calcium, ionized calcium, and sodium. Phosphorus, magnesium, and potassium concentrations decreased significantly in December at least compared to July levels for females. In males, significant variations were observed only for magnesium, evidencing higher values in March (median 3.5 mg/dL) and November (median 4.2 mg/dL) when compared to the other seasons (December and July medians of 2.9 and 3.0 mg/dL, respectively). Chlorine levels were constant for males, while females had significantly lower values in March (median value of 117.5 mEq/L), compared to the ones from December (134.0 mEq/L) and November (134.0 mEq/L).

PCA of biochemical data evidenced a general overlapping profile of December and November for males and females (Supplementary Figure S2). For males, March, July, and November are partially convergent (Supplementary Figure S2A), while for females, the three reproductive phases appear in different clusters (Supplementary Figure S2B). For both females and males, four factors account for 79.28% (PC1—32.67%, PC2—26.73%, PC3—11.52%, and PC4—8.36%) and 74.00% (PC1—32.34%, PC2—17.19%, PC3—14.83%, and PC4—9.64%) of data variance, respectively. The higher PC1 positive loadings were found with total protein, cholesterol, magnesium, and phosphorus, for females, and total protein, cholesterol, total calcium, and triglycerides for males.

### 3.4. Quantification of Plasma Hormones

**Figure 2:**
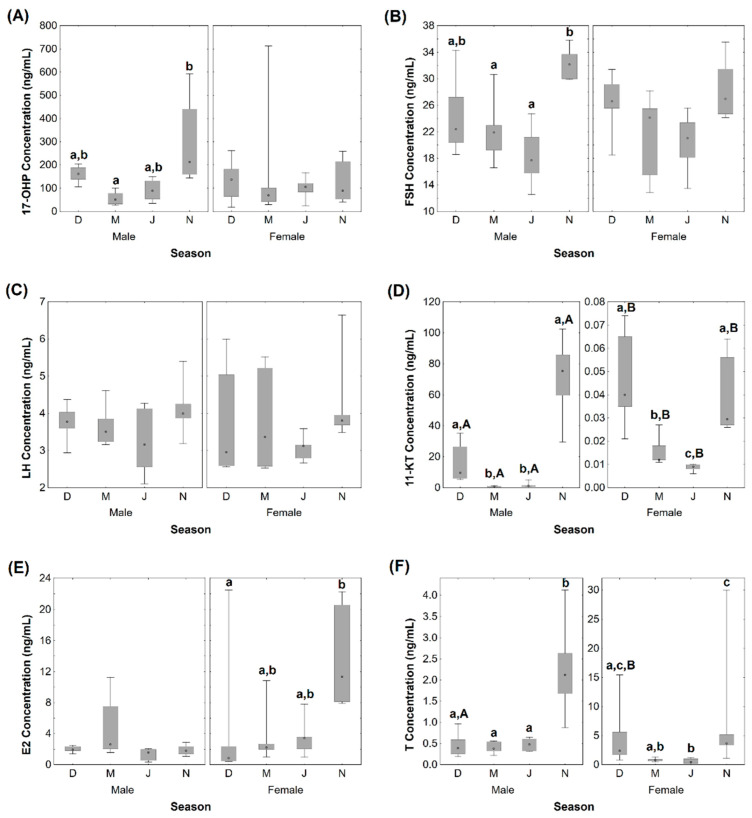
Plasma concentrations (ng/mL) of 17α-hydroxyprogesterone—17-OHP (**A**), follicle-stimulating hormone—FSH (**B**), luteinizing hormone—LH (**C**), 11-ketotestosterone—11-KT (**D**), estradiol—E2 (**E**), and testosterone—T (**F**) in male and female adult brown trout along the distinct reproductive phases (December, March, July, and November). Data are given as median (circle in the box), 25% and 75% percentile (upper and lower limits of box), and maximum and minimum values (in the whiskers); *n* = 6 animals/sex/season. Different lower-case letters mean significant differences (*p* < 0.05) among seasons within a sex. Different upper-case letters mean significant differences (*p* < 0.05) between sexes within a season.

Hormone levels along the four reproductive phases are shown, for both sexes, in Figure 2. The 17-OHP median levels for females were the highest in December (136.27 ng/mL), compared to March (68.53 ng/mL), July (104.85 ng/mL), and November (89.56 ng/mL). As for males, the highest median concentration was detected in November (212.45 ng/mL), and lower medians were observed in December (161.83 ng/mL), March (50.30 ng/mL), and July (88.44 ng/mL). Significant differences were only proven between males from March and November (Figure 2A). The FSH (Figure 2B) and LH (Figure 2C) levels were higher in November, for both males and females. Despite that, no significant alterations were observed for LH, while for males, FSH levels in November (median 32.14 ng/mL) differed significantly from the ones quantified in March (median 21.89 ng/mL) and July (median 17.68 ng/mL). For both males and females, the 11-KT concentrations were significantly higher in November (medians 75.35 and 0.030 ng/mL, respectively) and December (medians 9.56 and 0.040 ng/mL, respectively) than in the remaining seasons (Figure 2D). Also, for females, the 11-KT levels in March (median 0.012 ng/mL) were significantly higher than those obtained in July (median 0.009 ng/mL). Significant differences between males and females were found in all seasons, since females had much lower concentrations of 11-KT. E2 concentrations in males did not vary between seasons, but in females, the concentrations were significantly higher in November (median 11.34 ng/mL) compared to December (median 0.87 ng/mL) (Figure 2E). Overall, for both males and females, T concentrations were significantly higher in November (2.12 ng/mL and 3.64 ng/mL, respectively) in relation to March and July. In December, significant differences were observed between sexes (males—0.39 ng/mL vs females—2.36 ng/mL) (Figure 2F).

The PCA plot of plasma hormones revealed different overlapping patterns for females and males (Supplementary Figure S3). For males (Supplementary Figure S3A), the first two factors account for a total data variation of 78.49% (PC1—53.64% and PC2—24.85%), and PC1 is positively associated with 11-KT and T. For females (Supplementary Figure S3B), PCA exhibited two factors with eigenvalues greater than 1, which explained 69.16% of the variation in the data (PC1—47.04% and PC2—22.12%). E2 and T have the highest positive loadings on PC1.

### 3.5. Qualitative and Semi-Quantitative Grading of Gonadal Maturation Stages

**Figure 3:**
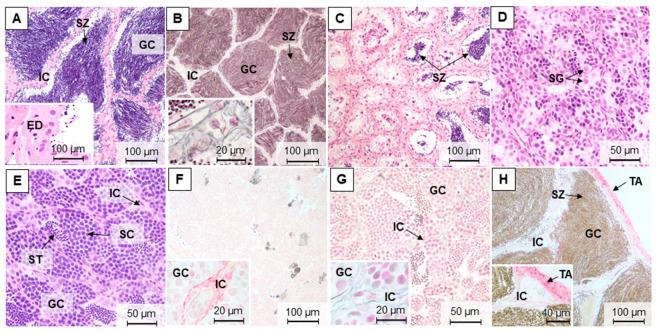
Representative methacrylate sections of adult brown trout testes in December (**A**,**B**), March (**C**), July (**D**–**G**), and November (**H**). Sections were stained with: H&E (**A**,**C**–**E**), Sirius red (**F**,**H**), and reticulin (**B**,**G**). IC—interstitial compartment; GC—germinal compartment; ED—efferent ducts; TA—tunica albuginea; SG—spermatogonia; SC—spermatocytes; ST—spermatids; SZ—spermatozoa.

During the four reproductive phases, it was possible to differentiate five maturation stages in brown trout testes. In late spermatogenic testes (stage 3), sampled in December and November, all germ cell stages were observed, but the spermatozoa were predominant (Figure 3A,B,H). In spent testes (stage 4), sampled in March, it was possible to observe testicular lobules containing residual spermatozoa (Figure 3C). The most undeveloped testes corresponded to stage 0, where gonads only displayed immature germ cells (Figure 3D), which were found in March and July. In the early spermatogenic phase (stage 1), sampled in July, it was possible to observe spermatozoa, but the immature germ cells were predominant (Figure 3E).

Testes sections stained with Sirius red showed that collagen fibers were marked with a reddish staining. Reticulin staining showed the reticular fibers (type III collagen) in black, after silver impregnation. Both fibers were in blood vessels, tunica albuginea, and within the testicular interstitium in all samplings. Overall, the marked patterns in the testis interstitium were more continuously distributed in July, either with Sirius red (Figure 3F) or reticulin staining (Figure 3G), compared to the other seasons (Figure 3B,H).

**Figure 4:**
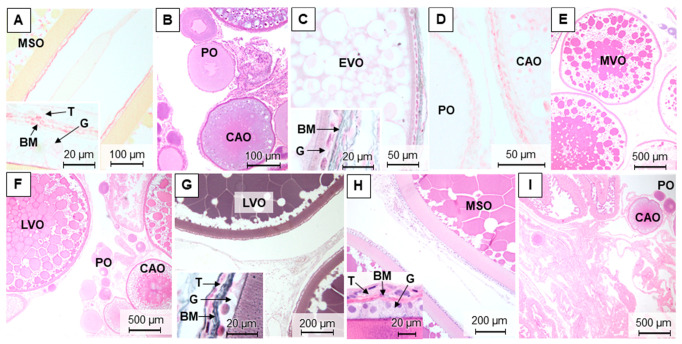
Representative methacrylate sections of adult brown trout ovaries in December (**A**), March (**B**–**D**), July (**E**–**G**), and November (**H**,**I**). Sections were stained with: H&E (**B**,**E**,**F**,**H**,**I**), Sirius red (**A**,**D**), and reticulin (**C**,**G**). G—granulosa; T—theca; BM—basement membrane; PO—perinucleolar oocytes; CAO—cortical alveolar oocytes; EVO—early vitellogenic oocytes; MVO—mid-vitellogenic oocytes; LVO—late vitellogenic oocytes; MSO—mature/spawning oocytes.

For brown trout females, it was possible to differentiate five maturation stages in the ovaries along the different samplings (Figure 4A–I and Supplementary Figure S4). In the most advanced maturation stage, the stage 4 or late development/hydrated phase, the ovary was mainly composed of late development and mature/spawning oocytes (Figure 4A,H). This stage was observed in December and November. On the contrary, the early development phase (stage 1) in March showed a predominance of perinucleolar and cortical alveolar oocytes (Figure 4B–D). Stage 2 ovaries, in March and mostly in July, were composed of mid-development oocytes that filled approximately half of the gonad (Figure 4E). In July, stage 3 corresponds to well-developed ovaries, and the late vitellogenic oocytes were the most abundant (Figure 4F). Right after ovulation (stage 5), there was an abundance of spent follicles, remnants of theca, and granulosa in the gonad (Figure 4I).

In the ovaries, there were no major differences in the fiber content between seasons as demonstrated when using both Sirius red (Figure 4A,D) and reticulin staining (Figure 4C,G).

**Figure 5:**
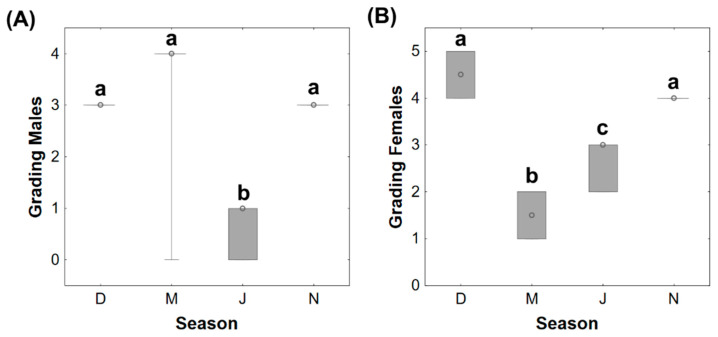
Male (**A**) and female (**B**) brown trout gonadal maturation stages along the distinct reproductive phases (D—December, M—March, J—July, and N—November). Ovary maturation stages: 1—early development; 2—mid-development; 3—late development; 4—late development/hydrated; 5—post-ovulatory. Teste maturation stages: 0—undeveloped; 1—early spermatogenic; 2—mid-spermatogenic; 3—late spermatogenic; 4—spent. Data are given as median (circle in the box), 25% and 75% percentile (upper and lower limits of the box), and maximum and minimum values (in the whiskers); *n* = 6 animals/sex/season. Different lower-case letters mean significant differences (*p* < 0.05) among sampling seasons within a sex.

Overall, the gonadal grading showed a higher gonadal maturation in December and November, compared to March and July, for both males and females (Figure 5A,B). For the testis (Figure 5A), there were no differences between grades in December (median 3), November (median 3), and March (median 4), but all differ significantly from July (median 1). In the ovaries (Figure 5B), higher grades were found in December (median 4.5) and November (median 4), compared with July (median 3) and March (median 1.5). Also, the ovaries from July had significantly higher grading than the ones from March.

### 3.6. Immunohistochemistry of Brown Trout Gonads

#### 3.6.1. CYP19

**Figure 6:**
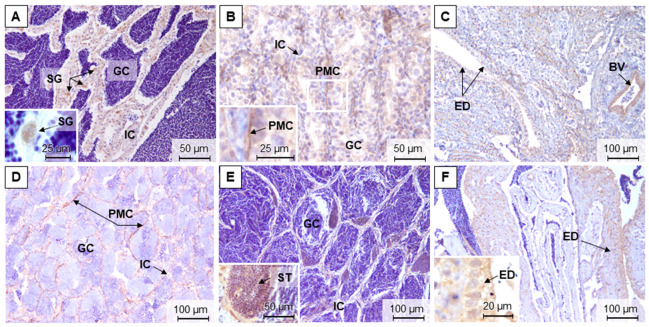
CYP19 immunoreactivity in adult brown trout testes in December (**A**), March (**B**,**C**), July (**D**), and November (**E**,**F**). GC—germinal compartment; IC—interstitial compartment; ED—efferent ducts; PMC—peritubular myoid cells; SG—spermatogonia; BV—blood vessels; ST—spermatids.

Male gonads displayed a moderate to strong staining of CYP19 in December, in both the spermatogonia and interstitium (Figure 6A). In March, the signal varied from light to intense within the interstitium, resulting from inter-animal variations. CYP19 positive staining in spermatogonia was also observed in March, but it was not as evident as in December. In March, staining of peritubular myoid cells was found sporadically (Figure 6B), along with an intense immunoreaction noted in blood vessels (Figure 6C), which was also present in the remaining seasons. In July, all animals exhibited an abundant and strong signal in the peritubular myoid cells (Figure 6D). In addition, in July the interstitial areas showed limited immunopositive signal, while no relevant stain was observed in spermatogonia. In November, the interstitial staining pattern was similar to the one found in December. Despite this, in November testes showed an exclusive CYP19 immunolabeling in spermatids (Figure 6E). Further, an intense immunoreaction was noted in the epithelium of efferent ducts (columnar and cuboidal cells) in all sampling seasons (Figure 6C,F).

**Figure 7:**
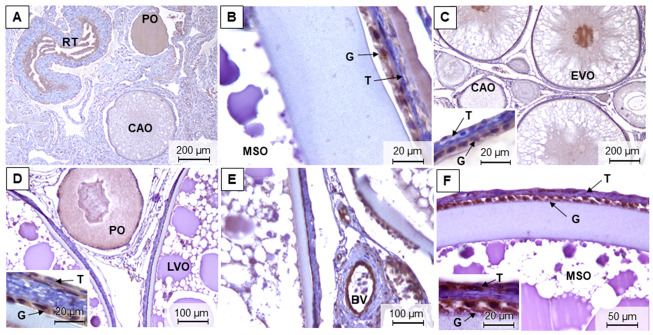
CYP19 immunoreactivity in adult brown trout ovaries in December (**A**,**B**), March (**C**), July (**D**,**E**), and November (**F**); G—granulosa; T—theca; PO—perinucleolar oocytes; CAO—cortical alveolar oocytes; RT—remnant gonad tissues; EVO—early vitellogenic oocytes; LVO—late vitellogenic oocytes; MSO—mature/spawning oocytes; BV—blood vessels.

In December, the female remnant gonads showed strong CYP19 immunolabeling in the epithelium and in the ooplasm of both primary and cortical oocytes (Figure 7A). In the granulosa and theca layers of late vitellogenic and mature/spawning oocytes, an intense positive staining was also observed (Figure 7B). Immunopositivity in the ovaries sampled in March and July had a similar pattern to the one in December, although the ooplasm of primary and cortical oocytes was marked less intensely (Figure 7C,D, respectively). Overall, in the ovaries, intense immunolabeling in the blood vessels was also observed (Figure 7E). In November, a stronger signal in the ooplasm of primary oocytes was noted, compared to December. The granulosa and theca layers of late vitellogenic and mature/spawning oocytes were also stained with more intensity than in December (Figure 7F).

There was no CYP19 immunostaining in testes and ovaries used as negative controls.

#### 3.6.2. CYP17-I

**Figure 8:**
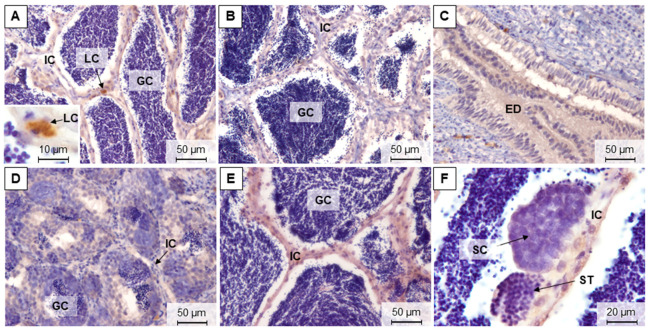
CYP17-I immunoreactivity in adult brown trout testes in December (**A**), March (**B**,**C**), July (**D**), and November (**E**,**F**). GC—germinal compartment; IC—interstitial compartment; ED—efferent ducts; LC—Leydig cells; ST—spermatids; SC—spermatocytes.

Overall, the CYP17-I immunoreactivity in testes was noted in the interstitial cells (likely Leydig cells), with a granular appearance in all four reproductive phases. In December, the signal was moderate in the interstitium (Figure 8A), while in March (Figure 8B), testes displayed less intense staining, as a consequence of a smaller number of interstitial cells. In all samplings, spermatogonia and Sertoli cells were sporadically stained, whereas the epithelial cells of efferent ducts were always marked (Figure 8C). July showed a similar staining pattern as March in the interstitium (Figure 8D). In November (Figure 8E), the reactivity in the interstitium was similar to December, but spermatocyte and spermatid clusters were also positively marked (Figure 8F).

**Figure 9:**
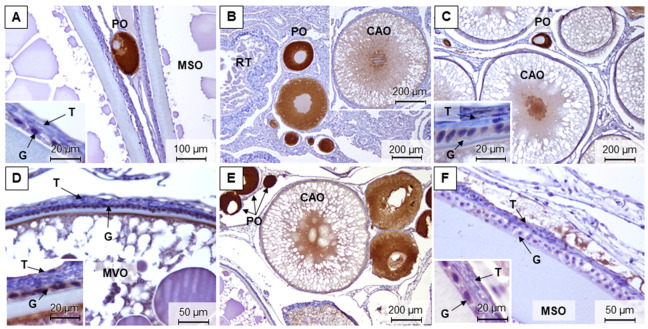
CYP17-I immunoreactivity in adult brown trout ovaries in December (**A**,**B**), March (**C**), July (**D**,**E**), and November (**F**). PO—perinucleolar oocytes; CAO—cortical alveolar oocytes; RT—remnant gonad tissues; MVO—mid-vitellogenic oocytes; MSO—mature/spawning oocytes.

Ovaries from December (Figure 9A) showed weak CYP17-I immunolabeling in the granulosa cells of late vitellogenic and mature oocytes. The females from December, with remnant gonads (Figure 9B), showed a very strong CYP17-I staining in the ooplasm of primary and cortical alveoli oocytes. The labeling increased in March (Figure 9C) and July (Figure 9D) when a light signal became visible in the theca layer. Primary and cortical alveoli oocytes also exhibited an intense signal in March, July (Figure 9E), and November. In November (Figure 9F), ovaries showed a similar signal pattern in granulosa cells of late vitellogenic and mature oocytes, as in December.

No CYP17 immunoreactivity was noted in the negative controls.

#### 3.6.3. 17β-HSD

**Figure 10:**
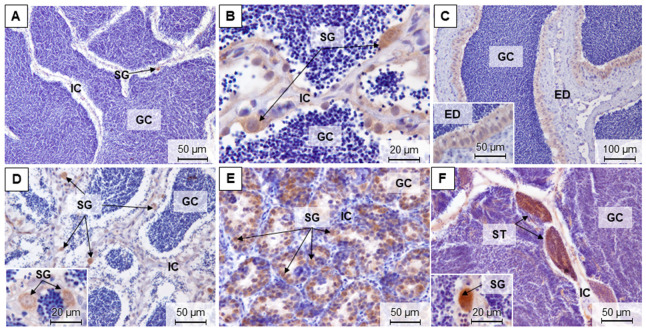
17β-HSD immunoreactivity in adult brown trout testes in December (**A**–**C**), March (**D**), July (**E**), and November (**F**). SG—spermatogonia; GC—germinal compartment; IC—interstitial compartment; ED—efferent ducts; ST—spermatids.

Testes from all seasons presented an intense 17β-HSD signal in spermatogonia. These cells were sporadic in December (Figure 10A,B). The columnar and cuboid epithelium cells of the efferent ducts exhibited a specific signal, in all samplings (Figure 10C). In March (Figure 10D) and July (Figure 10E) spermatogonia increased numerically. In November, spermatid clusters were also positive for 17β-HSD and the signal was more intense in spermatogonia (Figure 10F).

**Figure 11:**
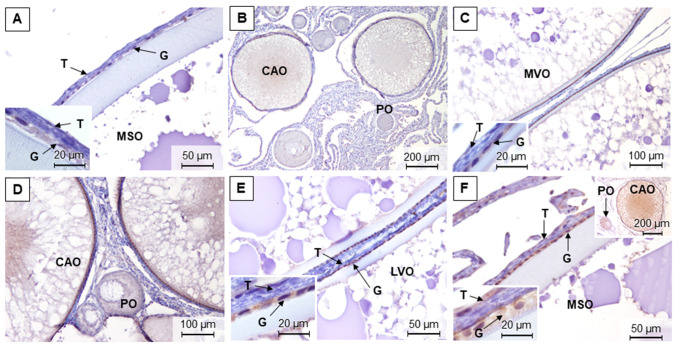
17β-HSD immunoreactivity in adult brown trout ovaries in December (**A**,**B**), March (**C**,**D**), July (**E**), and November (**F**). G—granulosa; T—theca; PO—perinucleolar oocytes; CAO—cortical alveolar oocytes; MVO—mid-vitellogenic oocytes; LVO—late vitellogenic oocytes; MSO—mature/spawning oocytes.

In December, the ovaries showed a light 17β-HSD reactivity in the granulosa cells (Figure 11A), and in the ooplasm of primary and cortical alveoli oocytes (Figure 11B). The immunostaining in the granulosa layer increased in March (Figure 11C), and the ooplasm staining of primary and cortical oocytes was similar to December (Figure 11D). In July, the signal also increased in the granulosa layer (Figure 11E), but in November, the signal was stronger for both granulosa cells (Figure 11F) and primary and cortical oocytes, compared to the other seasons.

Negative controls showed no 17β-HSD specific signal.

## 4. Discussion

### 4.1. Biometric Parameters

Our data confirm that brown trout spawns in Portugal between November and January, corresponding to the autumn/winter season, as it is mostly reported for the species [18,22]. During the developing and spawning-capable season (November and December), the total length and weight of individuals were lower compared with the remaining seasons. This can be explained by the fact that those two sampling months were in different years. We started the study in December with a 3-year-old fish cohort, which grew over the following months, and then we ended the assay by sampling a new 3-year-old cohort in November; with fish having a similar biometry to those from the previous year. In addition, in this study the fish sampling in the aquaculture tanks was random. Therefore, the calibration of fish was done considering only the age cohort and not the size of the specimens. In this vein, we emphasize that there is a natural variability between the breeding stock individuals, even if being of the same age and sampled at the same season.

Regarding the GSI, the maximum levels were reached during the developing stage (November), for both males and females. The gonad weight and the GSI peaked when gonads were filled with the later stages of spermatogenic or oogenic cells, confirming that spermiation and ovulation were close. The abrupt decrease of spermatozoa content, as observed here at the regressing stage, indicates the end of spermiation [33]. In males, the early spermatogenic stages and the lowest GSI values were found mainly in the regenerating stage (July), which is in line with previous brown trout data [22]. As for females, the GSI and gonad weight decreased significantly to minimum values in the regressing stage (March). The obtained GSI profile in the present study is in agreement with the development GSI phases described in female trout [34].

Seasonal changes in male and female liver weight showed significantly lower values during the spawning capable season (December). The increase in liver weight and HSI after this stage, particularly in females, reflects the production of several constituents, such as Vtg, which is synthesized in the liver and deposited into the gonad throughout vitellogenesis [35].

Concerning the changes in brown trout eggs, it has been reported that their maximum size is reached at the end of maturation and depends mainly on the female size [22]. During the developing and spawning-capable season (November and December), the number of eggs was the lowest, but eggs had significantly higher diameters when compared to the other seasons. This may indicate that several maturing oocytes degenerate, according to the oocyte atresia phenomenon already recognized in fish [36]. Other brown trout populations from Turkey [18] and Tibet [22] showed similar egg biometric parameters as the ones found here.

### 4.2. Biochemical Analyses

Fish reproductive processes in seasonal breeders are accompanied by variations in lipid metabolism [37,38]. Among the main plasma lipoproteins, HDL is generally the most abundant lipoprotein in trout plasma [11], explaining the values above the quantification limits obtained herein, which made it impossible to obtain a seasonal profile. The increased biosynthesis of sex steroid hormones along the reproductive cycle may lead to the decline in their precursor cholesterol, as noticed in December and November for both males and females. Although no significant differences were found for triglycerides, a decreasing tendency was noticed in December and November, at least in males. Other brown trout studies showed a drop of about 50% in the triglycerides during the reproductive season [39]. As for LDL, the lower levels were observed in November and December for both sexes, which may indicate the incorporation of this lipoprotein in the gonads. This phenomenon was described in male rainbow trout at the time of spermiation [37]. In female fish, lipid mobilization to the oocytes occurs by the incorporation of Vtgs and VLDL [40], however, we did not note changes in VLDL levels among reproductive phases. Vtg is the egg yolk precursor protein expressed mainly in oviparous vertebrate females and contains two major yolk proteins, lipovitellin and phosvitin [41]. Phosvitin is important in sequestering calcium, magnesium, and other minerals [6]. As the shift from vitellogenesis to the maturation stage occurs, brown trout stops requiring Vtg for oocyte incorporation. Therefore, the decrease observed in the phosphorus levels at developing and spawning-capable seasons (November and December) likely results from decreases in Vtg levels.

In general, the concentrations of ions in females were higher during vitellogenesis and lower in the spawning-capable season. Also, evidence shows that during hydration the intake of these ions into the oocyte also occurs, balancing osmotic pressure and allowing the water influx [42,43]. Ions were mostly stable during the male reproductive phases, except for magnesium which showed the highest levels in November. A similar range of calcium, magnesium, sodium, potassium, and chlorine values was exhibited in rainbow trout [44]. Reduced levels of glucose and total protein were observed during November and December when the gonads are mature and near spawning and spermiation. The findings contribute to the idea that the reductions may be explained by the consumption of proteins and glycogen stocks [45]. A decrease in the concentration of glucose at the time of spawning was noticed in rainbow trout [45] and English sole (*Parophrys vetulus* Girard, 1854) [46].

As for hemoglobin and hematocrit, no major changes were observed. The levels are comparable to those found in other trout studies [45,47].

### 4.3. Hormonal Concentrations

In rainbow trout, the present knowledge states that the gonadotropins FSH and LH are differently secreted along a reproductive cycle, with FSH mainly regulating vitellogenesis and spermatogenesis, and LH the latter maturation stages and ovulation or spermiation [48,49]. Accordingly, and although we only found statistical differences in brown trout males, the FSH plasma levels in both sexes had a maximum peak at the developing stage (November) and declined (in males) in the spawning capable season (December). For LH, the plasma levels were mostly constant and low along the four reproductive phases. Slightly higher concentrations were noted in November, which corresponded to the latter maturation stages of male and female gonads. A possible explanation for not having detected an LH peak in the spawning capable season (December) may have to do with the fact that some animals in November were already very close to spawning and spermiation. It should be noted that the implemented immunoassays were not species-specific, which can cause some misinterpretations in this type of result, but the fact is that other studies (without this constraint) reported low to no changes in LH plasma levels, not reflecting the observed increase in the mRNA relative contents [48,50]. Therefore, even in closely related fish, nuances may exist in gonadotrophin fluctuations. Also, it should be emphasized that, despite not being possible in this study, an increase in the number of animals and the inclusion of other sampling months, with the corresponding reproductive phases throughout the reproductive cycle, would be beneficial, as we may not have detected hormonal peaks, especially in the period of most rapid gonadal development. A future study to explore the rapid gonadal recrudescence occurring from August to October could be valuable.

In males, the concentration pattern of the androgens 11-KT and T followed the same trend as the one for FSH, increasing during the development of spermatogenesis (from March to November) and decreasing at the end of spermiation. The changes followed those evidenced in the coho salmon (*Oncorhynchus kisutch* Walbaum, 1792) testis [51]. It is reported in fish that FSH induces the synthesis of androgens (both 11-KT and T) by acting through the FSH receptor in the Leydig cells [52]. As in the coho salmon, brown trout males showed a predominance of 11-KT over T in all sampled seasons [3]. Both androgens are less effective in stimulating spermiation, compared to the progestin 17α,20β-DP (17α,20β-dihydroxy-4-pregnen-3-one), a maturation-inducing hormone [53]. The synthesis of 17α,20β-DP by the precursor 17-OHP is well-established in females [54], but its significant roles in male fish have been also reviewed [55]. In rainbow trout, the 17-OHP induced the production of 17α,20β-DP in sperm [56], which may explain here the maximum 17-OHP levels before spermiation in November.

Lokman, et al. [57] reported that 11-KT levels were detected in females from the Cyprinidae, Salmonidae, and Scombridae families, despite T being the predominant androgen [57]; which also happened here for brown trout. The plasma concentration of T was high in the developing stage (November) and then decreased during the spawning capable season (December), which is in agreement with rainbow trout data [2]. Regarding E2 concentrations in brown trout females, the synthesis from its precursor, T, increased during oocyte growth and declined in spawning, as previously described [58]. E2 levels in brown trout males did not vary significantly over the four phases of the reproductive cycle, although its roles in the regulation of spermatogonia mitosis, and consequent germ cell renewal have already been pointed out [59]. The steroidogenic shift that occurs in female fish, from the end of vitellogenesis to the spawning time, is well described as corresponding to a decline in E2 levels in parallel with an increase in 17α,20β-DP concentrations [60]. Gonadotropins stimulate the thecal cells to produce 17-OHP, which is converted within the follicular cells into 17α,20β-DP [40,54,59] in distinct teleosts, including salmonids [61]. This conversion may justify why we did not find significant variations in 17-OHP levels between the reproductive phases.

### 4.4. Immunohistochemistry

For CYP17 immunohistochemistry, an anti-CYP17a1 antibody that tackles both the 17α-hydroxylase and 17,20-lyase activities [31] was used. This antibody was formerly implemented in fish gonads [31,62,63]. Previously, two different CYP17 genes were identified, which encode the proteins CYP17-I and CYP17-II [64]. CYP17-I is associated with both hydroxylase and lyase activities, crucial for the production of E2 for oocyte growth, while CYP17-II is only related to the hydroxylase activity, establishing itself in the production of 17α,20β-DP during oocyte maturation [65]. Here, the CYP17-I immunoreactivity was observed mainly in the interstitial cells, likely Leydig cells, and sporadically in spermatogonia and Sertoli cells, highlighting their crucial role as steroid-producing cells. The presence of CYP17 in putative Leydig cells was earlier observed, for instance in rainbow trout [66], Japanese eel (*Anguilla japonica* Temminck and Schlegel, 1846) [67], and zebrafish (*Danio rerio* Hamilton, 1822) [68]. The brown trout male efferent ducts revealed strong immunoreaction in both columnar and cubic epithelial cells in all samplings. In rainbow trout testis, CYP17 positive cells were observed in greater distribution close to the intratesticular efferent ducts [66].

In females, a weak CYP17-I immunoreactivity in the granulosa cells and no recognizable signal in the theca cells was observed during the spawning-capable and developing season (December and November), which increased in March and July. Moreover, the ooplasm of pre-vitellogenic oocytes revealed intense immunoreaction, whereas a very weak signal was noted in the vitellogenic and mature oocytes. In zebrafish, a non-seasonal breeder, a CYP17-I immunopositive signal was found in both granulosa and theca cell layers of vitellogenic oocytes and in the ooplasm of pre-vitellogenic oocytes [68]. Here, the decrease in the immunoreactivity in the theca and granulosa cells during the last stages of development agrees well with the steroidogenic pathway shift from oocyte growth to maturation, in which the expression of CYP17-I should decrease while that of CYP17-II should rise.

CYP19 expression in brown trout testes was noted in the cytoplasm of putative Leydig cells, Sertoli cells, and spermatogonia, demonstrating the relevance of E2 in male gonads. Similarly, CYP19a1a was strongly expressed in the Leydig cells and in the germ cells of zebrafish [68,69]. In brown trout, the CYP19 reactivity in endothelial and efferent duct cells was also evident, in line with the data reported for rainbow trout [70]. Further, in the regressing (March) and mostly in the regenerating season (July), peritubular myoid cells in brown trout were immunopositive. We could not find comparable data from fish, but our data agree with the immunostaining location for aromatase found in black bear testis [71]. Interestingly, new myoid cells were reported to occur in rainbow trout at each reproductive cycle, which may explain our results [72]. As for females, CYP19 showed an intense signal in the granulosa and theca cells in late vitellogenic and mature oocytes. This is not surprising since the steroidogenic conversion of cholesterol into T occurs in the thecal cell layer, which then diffuses into the granulosa cell layer, where it is aromatized by CYP19 into E2 [61]. The strongest CYP19 immunopositivity in the ooplasm of primary oocytes occurred in the developing stage. This should occur because CYP19 activity is expected to decline upon reaching oocyte maturation and consequent beginning of the drop in E2 titers.

The 17β-HSD is also assumed as a critical enzyme engaged in the final stages of T and E2 biosynthesis in the gonads [73]. In this study, testicular 17β-HSD immunoreactivity was overall predominant in spermatogonia, suggesting that these cells may be involved in androgen synthesis, as reported in the spotted ray (*Torpedo marmorata* Risso, 1810) [74]. In females, the 17β-HSD reactivity was observed in the granulosa cells and in the ooplasm of primary and cortical alveoli oocytes, and decreased at the spawning capable season (December). Accordingly, the lowest expression of 17β-HSD1 was noted in vitellogenic and mature follicles in zebrafish [75].

### 4.5. Gonadal Histology

In the literature, there are few and scope limited histological descriptions that detail the gametogenesis of brown trout along a reproductive cycle, covering specimens in Chile (cultured 3-year-old females) [9], Italy (males and females from river Metauro) [19], and Iran (cultured 4-year-old female and males) [76]. In this study, distinct gonadal maturation stages in adult males and females were graded in five maturation stages.

Further, there were observed variations in the distribution of reticular and collagen fibers in the interstitial compartment of the brown trout testis. Specifically, collagen fibers showed a continuous marked pattern in the interstitium in July (regenerating season). The peritubular myoid cells have been referred to as being responsible for the synthesis of extracellular matrix in the interstitium [77], which correlates this last result to the higher aromatase immunostaining in these cells in July. Further investigations must be made to decipher this possible relationship, since a high variability in the staining of collagen fibers was noted in different fish within seasons. Previously, in *Pimelodus maculatus* (Lacepède, 1803) testis a distinct distribution of collagen fibers along the reproductive cycle was noticed, with a higher incidence in the spawning season [78], which does not seem to occur, at least so clearly, in brown trout. Ovary sections showed no apparent differences in terms of collagen content between seasons. Nevertheless, reticular fibers were clearly highlighted as an integral part of the basement membranes and stroma. In piranha fish (*Serrasalmus maculatus* Kner, 1858), the reticulin staining also enhanced the continuity of the basement membranes in several stages of the reproductive cycle [79].

## 5. Conclusions

Changes in biometric, biochemical, hormonal profiles, as well as in gonad morphology in brown trout from both sexes, allowed the discrimination of key identifying parameters. Total protein and cholesterol were, simultaneously and in both sexes, the biochemical parameters that best explain the overall variance throughout the distinct gonadal stages. 11-KT and T for males and E2 and T for females mostly explained the variations between reproductive phases. The immunophenotypic location of the steroidogenic enzymes demonstrated site-specific, seasonal, and individual variations. Four distinct gonadal phases along the brown trout reproductive cycle were assessed by using a multi-parameter panel, which can be used as a tool to support future field and experimental investigation in areas related to reproductive toxicology and endocrine disruption.

## Figures and Tables

**Table 1:** Physico-chemical parameters of water in each sampling.

Season	Physico-Chemical Parameters of Water						
	Temperature (°C)	pH	O_2_ (%)	NO_2_ (mg/L)	NO_3_ (mg/L)	NH_3_ (mg/L)	Hardness (°dGH)
December	7.55 ± 0.49	6.44 ± 0.20	95.05 ± 2.05	0	0	0	1 ± 0
March	9.80 ± 1.27	6.56 ± 0.08	95.80 ± 2.55	0.03 ± 0.04	0	0	1 ± 0
July	16.40 ± 0.00	6.38 ± 0.11	84.90 ± 1.27	0.05 ± 0.00	0	0	1 ± 0
November	9.20 ± 0.57	6.07 ± 1.70	93.20 ± 1.70	0	0	0	1 ± 0

Data are expressed as mean ± standard deviation (SD); *n* = 2 for each season.

**Table 2:** Biometric data of adult brown trout throughout the distinct reproductive phases (December, March, July, and November).

Gender	Season	Total Weight (g)	Total Length (cm)	Standard Length (cm)	Condition Factor (K)	Gonad Weight (g)	GSI (%)	Liver Weight (g)	HSI (%)
Females	December	660 ^a^ 440−880	38.5 ^a^ 34.0−43.5	35.0 31.5−40.0	1.11 1.02−1.32	142.00 ^a^ 81.78−162.61	19.62 ^a^ 16.90−23.78	5.57 ^a^ 3.79−18.18	0.86 0.68−2.07
	March	1280 ^b^ 880−2000	48.3 ^b^ 41.0−53.0	44.0 38.0−49.5	1.26 1.13−1.34	5.40 ^b^ 3.43−12.45	0.42 ^b^ 0.37−0.73	18.38 ^b^ 11.06−22.30	1.22 1.12−1.69
	July	1240 ^b^ 680−2180	46.0 ^b^ 39.5−54.5	42.3 36.0−50.0	1.23 1.10−1.35	20.64 ^c^ 8.95−62.83	1.52 ^c^ 1.01−2.88	17.80 ^b^ 9.62−22.51	1.22 1.03−1.63
	November	810 ^a,b^ 620−900	41.7 ^a,b^ 38.0−43.4	38.1 35.0−40.2	1.12 1.01−1.23	152.44 ^a^ 124.99−202.86	20.54 ^a^ 17.02−23.05	12.25 ^a,b^ 7.43−17.88	1.67 0.84−1.99
Males	December	750 ^a^ 680−980	40.5 ^a^ 39.4−43.5	36.4 ^a^ 35.5−40.0	1.14 ^a^ 1.05−1.20	32.04 ^a^ 21.82−41.38	4.18 ^a^ 2.92−5.91	8.05 ^a^ 5.55−9.56	1.02 0.82−1.20
	March	1300 ^a,b^ 800−1880	45.8 ^a,b^ 38.0−50.5	42.3 ^a,b^ 35.5−47.5	1.36 ^b^ 1.27−1.46	5.07 ^b^ 2.83−8.95	0.44 ^b^ 0.15−0.84	15.96 ^b,c^ 8.13−21.85	1.19 1.02−1.43
	July	1710 ^b^ 800−2040	52.3 ^b^ 41.0−54.5	48.3 ^b^ 38.0−50.5	1.23 ^a,b^ 0.75−1.44	3.80 ^b^ 0.64−11.21	0.29 ^b^ 0.08−0.55	16.69 ^c^ 8.39−20.87	0.99 0.77−1.60
	November	840 ^a^ 580−1320	40.8 ^a^ 36.5−48.5	37.5 ^a^ 33.5−44.5	1.19 ^a,b^ 1.05−1.28	36.19 ^a^ 25.30−47.04	4.26 ^a^ 3.56−5.59	9.35 ^a,b^ 7.35−12.89	1.10 0.97−1.31

GSI—gonadosomatic index; HSI—hepatosomatic index. Data are expressed as median and minimum–maximum; *n* = 6 animals/season/sex. Different lower-case letters represent significant differences (*p* < 0.05), by Tukey test, among seasons within a sex.

**Table 3:** Biochemical data obtained from total blood, plasma, or serum of adult brown trout along the distinct reproductive phases (December, March, July, and November).

Gender	Season	Chol (mg/dL)	Trigl (mg/dL)	LDL (mg/dL)	VLDL (mg/dL)	Chol/HDL	Glu (mg/dL)	Hb (g/dL)	Htc (%)	TProt (g/dL)	TCa (mg/dL)	ICa (mg/dL)	P (mg/dL)	Na (mEq/L)	Cl (mEq/L)	Mg (mg/dL)	K (mmol/L)
Females	December	197 ^a,c^ 175−274	115.0 93.8−168.0	77.2 ^a^ 40.4−145.8	23.0 18.8−33.6	2.0 ^a,c^ 1.7−2.7	94.8 ^a,b^ 88.1−140.0	N/A <5.0−5.6	38.0 28.0−51.0	3.7 ^a^ 3.2−4.4	14.8 10.7−18.9	6.1 5.2−6.9	10.9 ^a^ 7.9−12.3	151.0 149.0−158.0	134.0 ^a^ 129.0−139.0	2.9 ^a^ 2.5−3.2	2.5 ^a^ 2.0−3.1
	March	367 ^b^ 307−500	97.7 86.7−115.0	244.1 ^b^ 185.0−380.7	19.5 17.3−23.0	3.6 ^b^ 3.0−5.0	198.5 ^a^ 130.0−>600.0	N/A <5.0	46.5 36.0−64.0	5.2 ^b^ 4.7−5.7	13.4 10.7−17.3	5.8 4.8−7.6	13.9 ^a,b^ 12.0−16.2	144.5 106.0−163.0	117.5 ^b^ 76.0−131.0	3.6 ^b^ 3.1−4.0	3.4 ^a,b^ 2.8−4.6
	July	305 ^a,b^ 238−365	111.5 92.6−128.0	184.4 ^a,b^ 113.8−242.6	22.3 18.5−25.6	3.0 ^a,b^ 2.4−3.6	134.0 ^a^ 91.7−211.0	N/A <5.0−12.7	35.0 25.0−55.0	5.8 ^b^ 4.8−6.4	17.8 13.3−20.4	6.2 4.9−7.6	14.3 ^b^ 12.9−15.8	160.0 145.0−176.0	129.0 ^a,b^ 125.0−141.0	3.5 ^b^ 3.2−3.7	4.2 ^b^ 3.1−6.6
	November	189 ^c^ 145−252	109.0 85.2−142.0	65.7 ^a^ 26.2−122.6	21.8 17.0−28.4	1.9 ^c^ 1.4−2.5	83.1 ^b^ 57.8−114.0	N/A <5.0−11.6	42.0 23.0−50.0	3.4 ^a^ 3.2−4.7	15.2 12.7−19.2	5.9 5.0−6.3	11.3 ^a^ 10.6−11.8	154.0 148.0−157.0	134.0 ^a^ 126.0−136.0	3.3 ^a,b^ 2.6−3.5	3.3 ^a,b^ 2.2−3.5
Males	December	175 ^a^ 133−237	97.4 90.8−106.0	55.3 ^a^ 12.3−116.0	19.5 18.2−21.2	1.7 ^a^ 1.3−2.3	73.1 ^a^ 66.8−141.0	N/A <5.0	45.5 15.0−46.0	3.7 ^a^ 3.4−4.2	11.6 10.6−12.0	5.9 5.3−6.3	12.9 9.0−13.5	153.5 149.0−162.0	129.5 127.0−138.0	2.9 ^a^ 2.7−3.3	2.6 2.0−4.1
	March	421 ^b^ 272−488	111.0 104.0−117.0	297.8 ^b^ 150.2−365.8	22.2 20.8−23.4	4.2 ^b^ 2.7−4.8	185.0 ^b^ 113.0−200.0	N/A <5.0	46.5 40.0−51.0	5.3 ^b^ 4.6−5.8	13.5 12.8−14.8	6.7 6.2−7.0	15.4 12.6−16.0	154.5 137.0−156.0	124.0 105.0−131.0	3.5 ^b^ 3.3−4.1	3.3 2.9−3.9
	July	368 ^b,c^ 171−422	100.3 83.0−125.0	246.1 ^b,c^ 47.8−296.0	20.1 16.6−25.0	3.6 ^b,c^ 1.7−4.2	124.0 ^a,b^ 84.3−230.0	N/A <5.0−12.4	54.0 45.0−65.0	4.6 ^b^ 4.4−5.4	12.3 11.2−13.8	6.1 5.6−6.3	13.2 11.4−17.9	155.5 141.0−165.0	128.0 110.0−134.0	3.0 ^a^ 0.2−3.6	3.6 2.5−6.6
	November	231 ^a,c^ 132−393	91.5 79.5−122.0	112.3 ^a,c^ 13.1−267.6	18.3 15.9−24.4	2.3 ^a,c^ 1.3−3.9	81.5 ^a,b^ 63.7−149.0	N/A <5.0−13.4	47.0 42.0−55.0	4.4 ^a,b^ 3.9−5.5	10.9 10.8−13.9	5.9 4.4−6.1	12.1 10.1−14.1	154.5 153.0−157.0	127.5 122.0−131.0	4.2 ^b^ 3.4−4.8	2.3 2.0−3.1

Chol—cholesterol; Trigl—triglycerides; LDL—low-density lipoprotein; VLDL—very low-density lipoprotein; HDL—high-density lipoprotein; Gluc—glucose; Hb—hemoglobin; Htc—hematocrit; TProt—total protein; TCa—total calcium; ICa—ionized calcium; P—phosphorus; Na—sodium; Cl—chlorine; Mg—magnesium; K—potassium. Data are expressed as median and minimum–maximum; *n* = 6 animals/season/sex. Different lower-case letters represent significant differences (*p* < 0.05), by Tukey test, among seasons within a sex.

## Data Availability

To preserve misuses, the data presented in this study are available only on request from the corresponding author.

## Supplementary Materials

The following are available online at https://www.mdpi.com/article/10.3390/ani11051290/s1. Figure S1: Principal component analysis scatter plot for male (A) and female (B) biometric parameters, Figure S2: Principal component analysis scatter plot for male (A) and female (B) biochemical parameters, Figure S3: Principal component analysis scatter plot for male (A) and female (B) hormonal parameters, Figure S4: Representative paraffin sections of adult brown trout ovaries stained with H&E at: stage 5—post-ovulatory (A) and stage 4—late development/hydrated (B), in December; stage 3—late development (C), in July; stage 2—mid-development (D) and stage 1—early development (E), in March. PO—perinucleolar oocytes; SF—spent follicles; CAO—cortical alveolar oocytes; EVO—early vitellogenic oocytes; MVO—mid-vitellogenic oocytes; LVO—late vitellogenic oocytes.
