# Supplementary figures and images for: Multi-Parametric Portfolio to Assess the Fitness and Gonadal Maturation in Four Key Reproductive Phases of Brown Trout
Source: Animals (Basel). 2021 Apr 30;11(5):1290. doi: 10.3390/ani11051290 (PMC8146139; doi:10.3390/ani11051290)

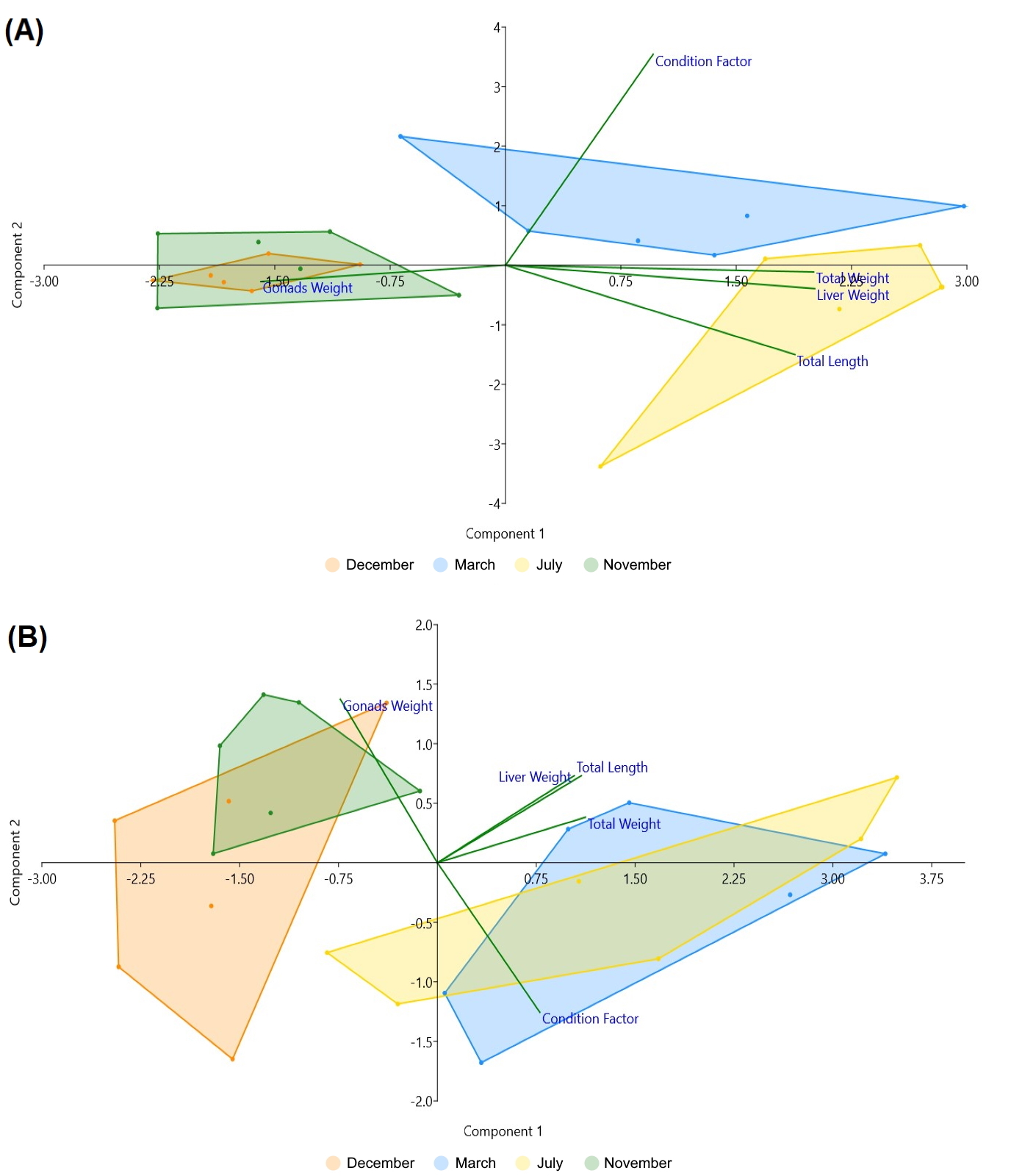

Supplement: Supplementary file 1 [file animals-11-01290-s001.zip › Supplem/Figure_1AB_SuppInfo.jpg]

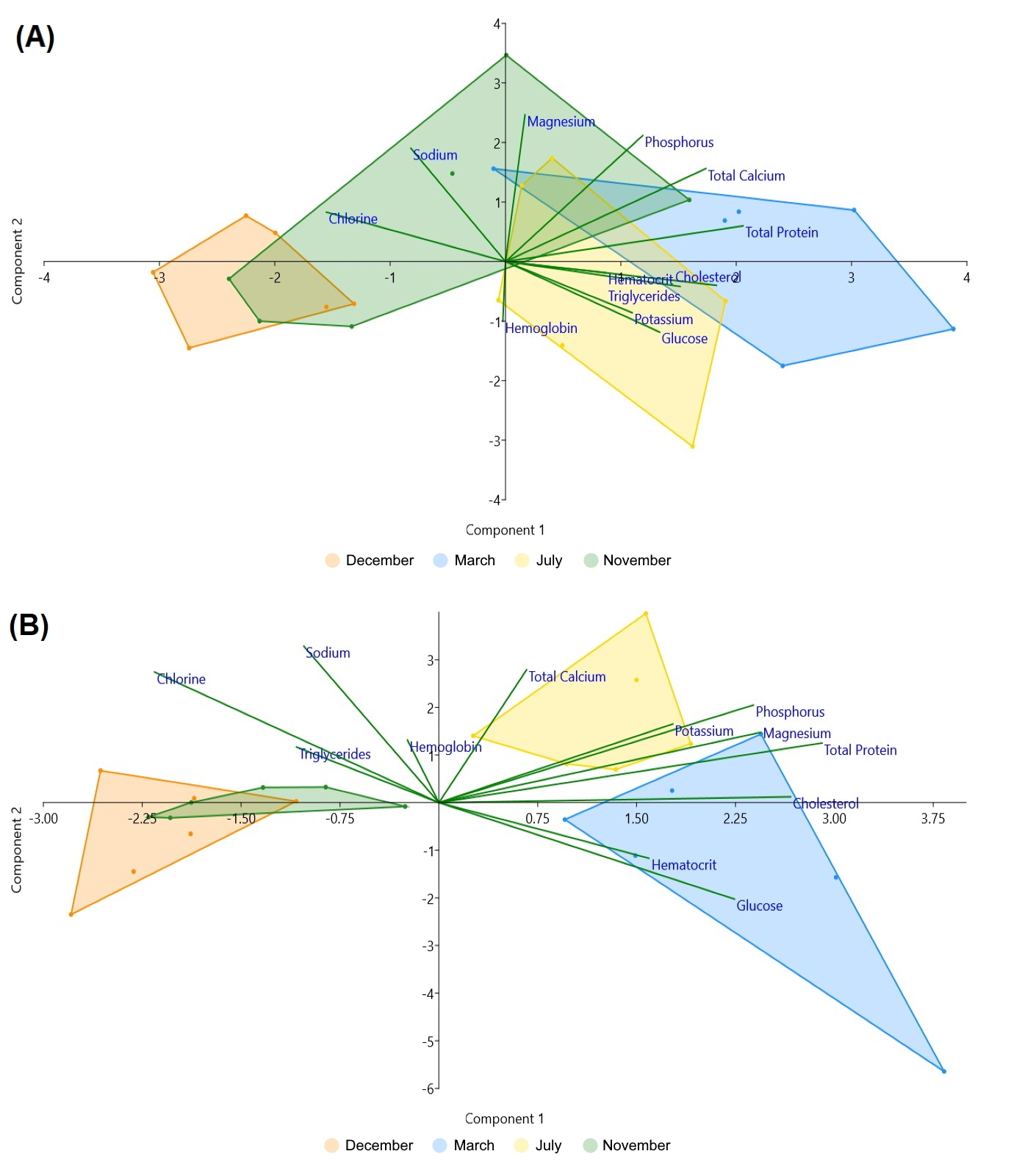

Supplement: Supplementary file 1 [file animals-11-01290-s001.zip › Supplem/Figure_2AB_SuppInfo.jpg]

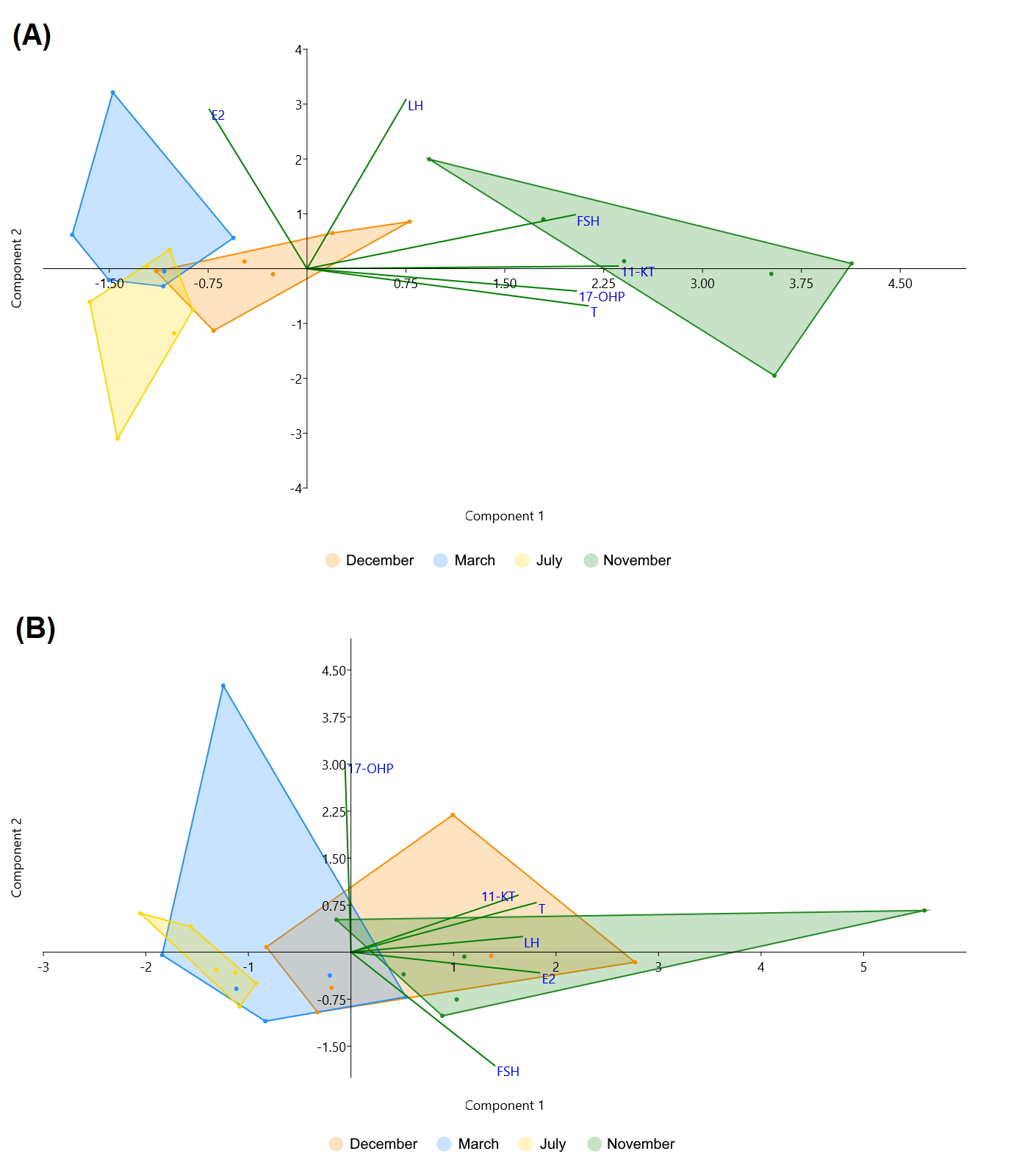

Supplement: Supplementary file 1 [file animals-11-01290-s001.zip › Supplem/Figure_3A_SuppInfo.jpg]

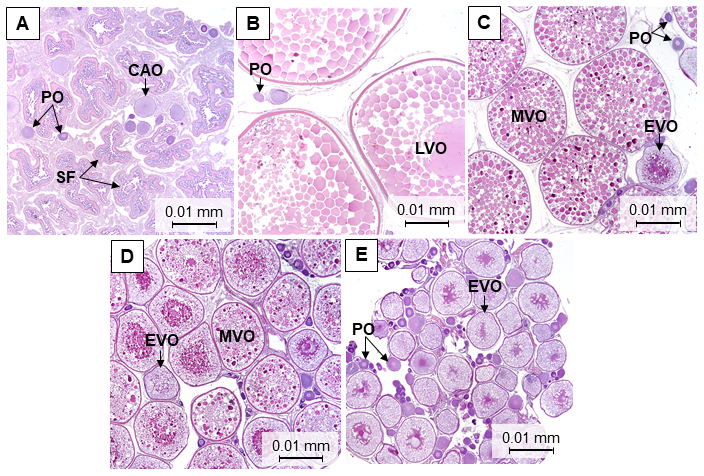

Supplement: Supplementary file 1 [file animals-11-01290-s001.zip › Supplem/Figure_4_SuppInfo.tif]
